# Critical Care and Surgical Management of Vascular Complications in Minimally Invasive Urological Reconstructive Surgery

**DOI:** 10.3390/jcm14196740

**Published:** 2025-09-24

**Authors:** Lucía Polanco-Pujol, Jorge Caño-Velasco, Rui Miguel Duarte Pedrosa, Claudia Fernandes, Luis López-Fando

**Affiliations:** 1 Department of Urology, Hospital General Universitario Gregorio Marañón, 28007 Madrid, Spain; 2 Department of Urology, ULS Coimbra, 3004-561 Coimbra, Portugal; 3 Department of Urology, ULS São João, 4200-319 Porto, Portugal; 4 Department of Urology, Hospital Universitario de la Princesa, 28006 Madrid, Spain; llfando@gmail.com; 5 UROLF Clinic, 28002 Madrid, Spain; 6 Catedra de Uroginecología, Universidad Alfonso X, 28691 Madrid, Spain

**Keywords:** vascular, complication, sacrocolpopexy, artificial urinary sphincter

## Abstract

**Background**: Despite the benefits of minimally invasive pelvic floor reconstructive surgery, serious life-threatening complications have been described. The most serious complications are vascular and intestinal. This review discusses the incidence, diagnosis, management and prevention of vascular complications in minimally invasive pelvic floor reconstructive surgery (sacrocolpopexy and artificial urinary sphincter). **Objectives**: We aimed to determine the incidence and management of vascular complications in minimally invasive pelvic floor reconstructive surgery. **Methods**: This narrative literature review on the incidence and management of vascular complications at sacrocolpopexy and artificial urinary sphincter was performed after the search of relevant manuscripts indexed in PubMed, EMBASE and Scielo published in English between January 2011 and June 2025. The keywords used were “vascular”, “complication”, “sacrocolpopexy”, and “artificial urinary sphincter”. We selected 19 manuscripts for comprehensive review. **Conclusions**: Dissection of the sacral promontory during sacrocolpopexy requires an exquisite knowledge of pelvic anatomy and adequate preoperative planning to avoid vascular injuries and find alternatives for mesh fixation if it cannot be performed in the usual anatomical location.

## 1. Introduction

**Figure 1:**
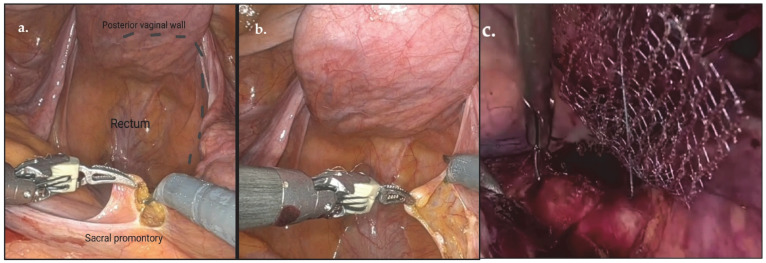
Step of sacrocolpopexy: (**a**) Access to sacral promontory; (**b**) dissection rectovaginal of the space; (**c**) mesh fixation. Source: Images owned by the author.

Sacrocolpopexy (SC) is the gold standard treatment for repairing pelvic organ prolapse. It must be performed in a regulated manner. The essential steps of SC include the following:

(a) Access to the sacral promontory: a peritoneal incision is made over the promontory, followed by dissection of the pelvic spaces, including the vesicovaginal and rectovaginal compartments, as shown in Figure 1.
(b) Mesh placement: polypropylene mesh is sutured to the anterior and posterior vaginal walls and vaginal dome.
(c) Mesh fixation: the mesh is elevated and redirected toward the sacral promontory.
(d) Peritoneal closure: the mesh is covered to ensure its integration into the retroperitoneal space.

Laparoscopic and robotic procedures are both considered minimally invasive surgical techniques. These approaches show significant advantages in reducing perioperative complications, allowing for better visualization of the anatomy. Moreover, increased surgical experience and high procedural volume are associated with improved outcomes and lower complication rates.

Intraoperative complications may involve injury to adjacent organs, such as the bladder, vagina, ureter, digestive system and vascular structures. The highest risk of complication is vascular injury, particularly for the middle sacral vessels during promontory dissection or to the iliac vessels. Although rare (<1%), these lesions may occasionally require conversion to an open technique. Additionally, the use of electrical energy for tissue dissection in laparoscopic and robotic surgery can result in occult injuries, which may not become clinically apparent until days or even weeks postoperatively.

SC presents notable challenges for surgeons, requiring careful and meticulous navigation through complex abdominopelvic vascular and neural structures that supply the pelvis, rectum and ureters. A thorough understanding of pelvic anatomy is essential to minimize the risk of potentially life-threatening complications.

The aim of this review is to determine the incidence and treatment of vascular complications in minimally invasive reconstructive surgery of the pelvic floor. The focus is mainly on SC as it is the most representative surgery.

## 2. Materials and Methods

A narrative literature review was conducted through a comprehensive search of PubMed, EMBASE and Scielo databases, between January 2011 and June 2025. Only studies published in English were included. The keywords used for the search were “vascular”, “complication”, “sacrocolpopexy”, and “artificial urinary sphincter”. The strategy involved examining all sections of the articles, including titles, abstracts, keywords and full texts. The reference lists of relevant articles were also reviewed to identify any additional sources that might have been missed in the initial search.

The inclusion criteria for this review encompassed: (1) studies assessing the incidence of complications associated with SC or artificial urinary sphincter (AUS) procedures; (2) studies specifically reporting vascular complications in the context of minimally invasive surgery; and (3) publications discussing techniques for preventing and treating complications.

Exclusion criteria were defined as follows: (1) studies focusing exclusively on vaginal or open abdominal approaches; (2) articles without accessible full text; (3) studies lacking data on laparoscopic or robotic techniques; and (4) publications deemed irrelevant to the scope of this review.

There was no limitation on the type of article (controlled trials, observational studies, systematic reviews, narrative reviews, meta-analyses, case reports, and letters to the editor). In the case of repeated experiences, the largest and most recent series was chosen.

**Figure 2:**
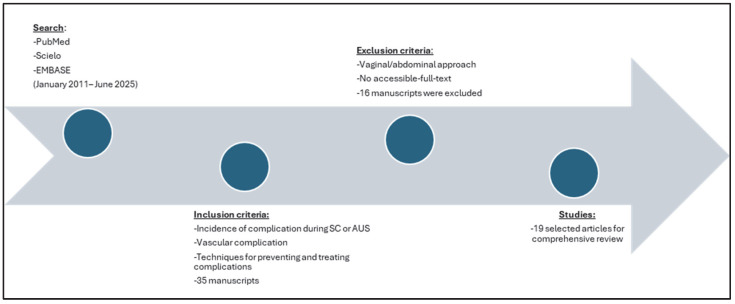
Flow chart of study selection in narrative review.

From an initial selection of 35 articles, 16 were subsequently excluded. Finally, we summarized 19 key manuscripts for narrative review. Data on the summarized articles are presented in Table S1 of the Supplementary Materials. The flowchart can be seen in Figure 2.

## 3. Results

The incidence of serious complications during SC is low in most series [1,2,3]. Although vascular complications are rare (0.8–1%), they are associated with the highest morbidity and mortality. Hemorrhage is the most common vascular complication [4]. Some authors include it as a complication, although they do not specify the type of underlying vascular injury [2].

The rate of intraoperative bleeding in minimally invasive pelvic floor surgery is low. Significant bleeding during surgery (≥500 mL) is rare, and the rate of transfusion during the perioperative period is less than 0.5%. Compared to laparoscopic sacrocolpopexy (LSC), robotic sacrocolpopexy (RSC) appears to result in significantly less blood loss. However, the clinical relevance of this is limited. Thus, the meta-analysis by Yang et al. [5] found no significant differences in perioperative transfusion rates or intra- and postoperative complications when comparing the two surgical approaches. In this meta-analysis, the most common complication was intraoperative bladder injury (2–3%), while the least common were intestinal (0.6–1%) and ureteral (0–1%) injuries. Other groups have reported similar results on complication rates [6].

The data referring to studies from a narrative review presenting perioperative vascular complications are summarized in Table 1.

Siddiqui et al. [7] compared RSC (*n* = 125) with abdominal access (*n* = 322), obtaining similar symptomatic and anatomical success, with no differences in vascular complications and transfusion rates (0.8% RSC transfusion rate). There were no cases of significant haemorrhaging, defined as estimated blood loss of more than 1000 mL. The estimated blood loss was 90.0 ± 89.3 mL.

Similarly, Nosti et al. [2] compared abdominal and minimally invasive SC, finding that the former had a higher overall complication rate (20.0% vs. 12.7%; *p* = 0.001), though there were no differences in anatomical outcomes. Focusing on minimally invasive approaches, LSC was associated with a higher complication rate (18% vs. 7%; *p* < 0.02) and greater blood loss than RSC.

In their randomised controlled trial comparing RSC and LSC, Anger et al. [8] described two serious vascular complications, with one case of left iliac vein transection occurring in each group. This study found no statistically significant differences in blood loss or intraoperative complications between groups. Unger et al. [9] describe similar rates of vascular injury (0.7%) due to bleeding from the right hypogastric vessel or presacral space. Therapeutic management consisted of primary intraoperative vascular closure and applying manual pressure with a sponge or vascular clamps, respectively. In their series, the conversion rate to open access was 1.9%; presacral hemorrhage (*n* = 1) and the presence of a right common iliac artery aneurysm (*n* = 1) represented two cases of access change. RSC was associated with a higher rate of estimated blood loss (greater than 500 mL) (2.5%) [9].

The mesh fixation method can also cause complications. Gutzeit et al. [10] describe a case of common iliac vein thrombosis caused by local anatomical changes resulting from non-absorbable tackers placement. The patient was diagnosed on the third postoperative day and required anticoagulant therapy and endovascular treatment involving the removal of the thrombus and placement of a stent.

Some studies [2,11,12] have compared complication rates in patients of normal weight and those with obesity. Surgical times and blood loss appear to be greater in overweight patients, likely due to the added surgical difficulty and altered anatomy. An abundance of pre-sacral fatty tissue could complicate the identification of different vascular elements in overweight and obese women, increasing the risk of a major vascular injury. In this context, minimally invasive surgical techniques appear to offer distinct advantages over open approaches. These include reduced intraoperative bleeding, lower conversion rates to open surgery, and enhanced ergonomic conditions for the operating surgeon. Nevertheless, these assumptions must be interpreted with caution. While anatomical challenges in overweight and obese patients may theoretically increase surgical complexity and risk, observational data such as that presented by Mahoney et al. [11] did not demonstrate significant differences in intraoperative complication rates—including bladder, intestinal, ureteral, or vascular injuries—across BMI categories. Similarly, Zhao and Martin [13], despite reporting an average BMI of 28.3 kg/m^2^, observed low rates of both intraoperative and postoperative complications in their analysis of RSC. These findings suggest that surgical outcomes may be more closely related to technique and surgeon experience than to patient BMI alone.

**Table 1:** Vascular complications in s: pooled data from clinical studies and case reports.

Study	Design	N	Surgical Approach	Vascular Injury (*n*)	Transfusion Rate (*n*)	Bowel Injury (*n*)	Ureteral Injury (*n*)	Bladder Injury (*n*)
Nosti et al. [2]	R	535	RSC and LSC	1	0	4	0	10
Siddiqui et al. [7]	R	125	RSC	1	1	0	NA	2
Anger JT et al. [8]	RCT	78	RSC and LSC	2	NA	2	NA	NA
Unger et al. [9]	R	370	RSC and LSC	3	2	6	0	4
Gutzeit, O. et al. [10]	CR	1	LSC	1	0	NA	NA	NA
Zhao and Martin [13]	R	47	RSC	0	1	0	1	2

LSC: laparoscopic sacrocolpopexy; RSC: robotic sacrocolpopexy; NA: not available; R: retrospective study; CR: case report; RCT: randomized controlled trial.

There are very few articles referring to vascular complications during other minimally invasive pelvic floor surgeries, such as AUS implantation. While revision surgery and AUS removal are both around 10–15% of cases, bleeding and hematoma complications are rarely the primary cause [14]. In many cases, postoperative hematoma is common but not clinically relevant, so its frequency in the literature is likely to be underdiagnosed. In a comparative study by Peyronnet et al. [15], outcomes between open and robot-assisted AUS implantation were evaluated. The robotic approach was associated with a significantly lower rate of postoperative complications (25% vs. 75%; *p* = 0.02), a non-significant trend toward fewer intraoperative complications (37.5% vs. 62.5%; *p* = 0.25), and reduced intraoperative blood loss (17 mL vs. 275 mL; *p* = 0.22). Continence outcomes were comparable between groups (75% vs. 68.8%; *p* = 0.75), suggesting that robotic assistance may offer perioperative advantages without compromising functional efficacy. Yip et al. [16] described an exceptional case of deep vein thrombosis associated with the compressive effect of the balloon reservoir. The patient developed thrombosis of the iliac and external femoral veins within 48 h postoperatively. Despite anticoagulation therapy with enoxaparin and warfarin, no clinical improvement was observed after three weeks, necessitating surgical removal of the reservoir and subsequent ectopic repositioning.

## 4. Discussion

SC is the standard surgical option for pelvic organ prolapse; the LSC and RSC approaches showed similar results, lower complication rates, and shorter hospital stays than the open approach [2,7]. However, there are few published studies that reflect perioperative vascular complications.

- Sacral promontory: anatomical and risk factor considerations.

During SC, identification of the sacral promontory is essential to fix the mesh to the anterior longitudinal ligament at the S1 level. In 75% of patients, the presacral nerve plexus is located to the left of the midline; therefore, dissection of the promontory should be approached from the right, just medial to the right edge of the right iliac artery. Bleeding from presacral vessels is one of the most worrying intraoperative complications and can be life-threatening. At this level, vital structures such as the bifurcation of the iliac artery, the right ureter, and the bifurcation of the abdominal aorta and inferior vena cava (IVC) cranial to the sacral promontory are located. The left common iliac vein is the structure most at risk for injury, as it can be less than 1 cm from the midline. The right common iliac artery is usually located 1.5–4 cm from the promontory and the middle sacral artery is located 0.2 ± 3.9 mm to the left of the mid-sacral promontory and 48.0 ± 15.4 mm below the aortic bifurcation. Studies conducted on cadavers show that the middle sacral artery is present in almost all cases, with an average diameter of 2 mm (range 1–4 mm). In most cadavers (61%), it was found to the left of the midline of the promontory, and in 30% to the right side, with an average distance of 4 mm (range 0–15 mm). Its proximity to the mesh attachment site increases the risk of intraoperative damage, which can result in significant bleeding [17,18,19].

**Figure 3:**
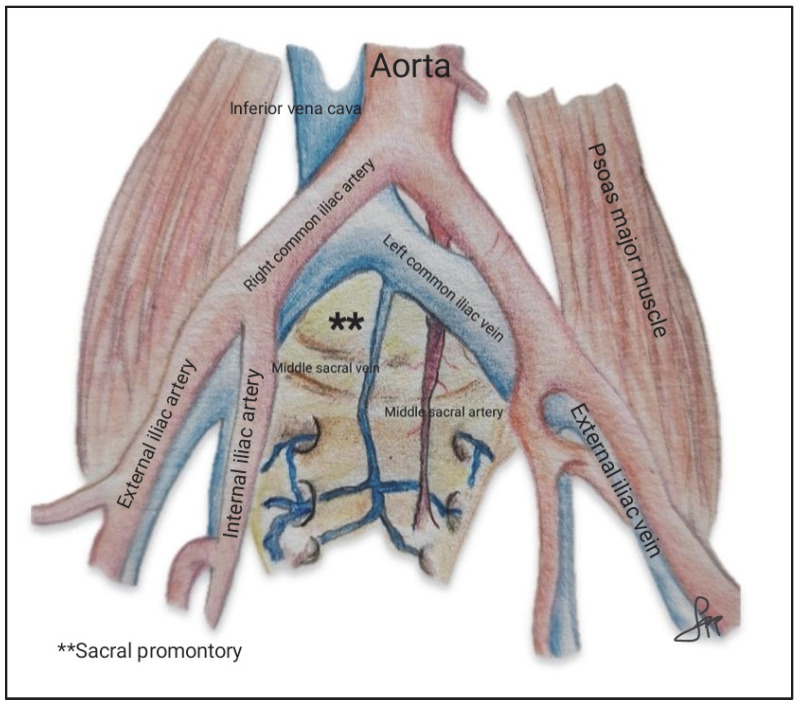
Vascular anatomy of the sacral promontory, highlighting the bifurcation of the abdominal aorta, the common iliac vessels, and the middle sacral artery and vein. The sacral promontory is marked (**). Source: Author’s own elaboration.

Identification of these vascular structures is crucial to prevent inadvertent injuries (Figure 3).

Identification of anatomical variants and numerary vessels is not uncommon [17,19]. Some authors have attempted to predict anatomical variants [18], recommending, in cases with abundant vascularization at the promontory, securing the mesh in a more secure alternative region. To reduce vascular risk at the time of mesh fixation, preoperative imaging planning models have been proposed. Berger et al. [20] used computed tomography (CT) scans to obtain regional anatomical measurements in two and three dimensions (3D). Their findings revealed that thin and elderly patients exhibited lower IVC bifurcations, located at or below the L5–S1 disc space, which may increase the risk of serious vascular injury during pelvic procedures. Despite these anatomical variations, no significant differences were observed in the accessible surface area for suture placement in the 3D models. Building on the relevance of age-related anatomical and surgical considerations, other recent studies have investigated age as a potential risk factor for perioperative complications. In this context, Carracedo et al. [21], analyzing a cohort of 123 RSC cases, concluded that the robotic approach is at least as effective and safe in women aged 70 years or older as it is in younger individuals, showing no increase in intraoperative or postoperative complication rates and achieving comparable anatomical and subjective success. These results may support wider implementation of minimally invasive SC among older patient populations.

Magnetic resonance imaging (MRI) and CT scans have also been used to characterise the relationship between the sacral promontory sutures and anatomical and vascular structures. To this end, Crisp et al. [22] placed vascular clips at the base of the sacral suture during RSC, with imaging performed at six weeks. The researchers observed that the left common iliac vein was located an average of 26 mm from the sacral suture, the right common iliac artery an average of 18 mm, and the right internal iliac artery an average of 10 mm. This study aimed to highlight the importance of adequate exposure and careful dissection of the promontory.

- Preventing Perioperative Bleeding: surgical approaches and alternative strategies.

Intraoperative bleeding rate is considered an indicator of surgical quality. Both LSC and RSC are minimally invasive techniques that consistently demonstrate lower blood loss compared to traditional open surgery, which is now largely considered obsolete due to technological advancement.

Degirmenci et al. [23] compared 3D LSC with traditional 2D LSC, concluding that blood loss was significantly higher in the 2D group, suggesting a benefit in bleeding rates due to the improved 3D view. Nevertheless, RSC is currently experiencing notable growth. Despite its higher cost and steeper learning curve, robotic platforms offer superior instrument articulation and high-definition 3D imaging, enabling precise dissection of critical anatomical landmarks such as the sacral promontory. These technical advantages contribute to reduced intraoperative bleeding and enhanced surgical performance, particularly when anatomical complexity or surgical precision is critical.

Other, more recently developed techniques involve the use of indocyanine green (ICG). Jun HS et al. [24] describe the cystoscopic intraureteric instillation of 5 cc ICG in RSC prior to surgery, followed by the intravenous ICG injection of 3 cc during presacral dissection and mesh fixation. This enabled the middle sacral artery to be clearly identified in 80% of patients, the superior hypogastric nerve in 73.3% of patients, and the right ureter in all cases, thus avoiding injury to these structures. The mean time from ICG injection to visualisation of the middle sacral artery was 43.7 s, and the mean duration of the arterial phase was 104.3 s. No cases of transfusion, ureteral injury, or intestinal or urinary dysfunction were documented in this study. Although this is a small-scale study, using ICG could help visualise anatomical structures in complex cases, with the aim of avoiding inadvertent injury.

Another strategy to avoid vascular complications is to make an incision in the peritoneum on the right side, over the sacral promontory [25]. The peritoneum should then be elevated to create a safe distance from the vascular structures. It is essential to identify the median sacral artery and vein, along with the sacral venous plexus, when operating in this space to avoid excessive bleeding. Wide dissection over the promontory is recommended to allow adequate identification of the vascular structures.

When significant vascularity is on the sacral promontory, surgeons should consider an alternative site for mesh attachment. Dubuisson et al. [26] first described laparoscopic lateral suspension (LLS) in 1998; in this procedure, the mesh is fixed laterally to the fascia of the abdominal wall. In Dubuisson’s study, the success rate was 86% and the recurrence rate was 4.6% at an 18-month follow-up. LLS is an effective and safe alternative technique for treating apical and anterior vaginal wall prolapse. Although LSC is the gold standard treatment for apical prolapse, it requires greater surgical expertise than lateral suspension. Sacral promontory dissection is complex and can lead to life-threatening complications, so this technique reduces perioperative risks by avoiding sacral promontory dissection, maintaining the physiological anatomy of the uterus and vagina in their natural position. Recent studies show anatomical and functional results similar to those of LSCP, with the advantage of better preservation of the vaginal axis and the possibility of performing it in cases of hysteropexy [27,28,29].

On the other hand, there is controversy regarding the method of attaching the mesh to the sacral promontory and its relationship to the risk of vascular complications. Studies comparing the use of non-absorbable sutures with spiral staples have been published, demonstrating that sutures offer greater resistance than staples [30]. The use of sutures is technically complex and requires more extensive dissection of the sacral promontory than staples; this may increase the risk of vascular injury, although it has a lower rate of spondylodiscitis due to its lower bone penetration.

- Management of complications.

The treatment for presacral vascular lesions varies depending on the type and severity of the lesion, as well as the patient’s haemodynamic stability (Table 2). If the patient is haemodynamically stable and the bleeding vessel is visible, direct haemostasis can be achieved using bipolar energy or by placing a surgical clip. However, direct coagulation carries the risk of increased bleeding when the vessels retract. Alternatively, orthopaedic tacks [31], bone wax or sutures can be used. Conservative treatment with haemostatic agents (such as Surgicel^®^, Fibrillar™ or FloSeal^®^) is also often useful [32]. In cases of major vascular injury or haemodynamic instability, haemostatic sutures or conversion to open surgery may be necessary [33].

- Other reconstructive surgeries.

Other minimally invasive reconstructive surgeries, such as the AUS, have a low rate of vascular complications. Most meta-analyses and systematic reviews report complications related to mechanical failure of the AUS [14,34,35]. Vaginal or urethral erosions (4–5%), infection (6–10%), bladder injury and surgical revision are the most common complications.

Vascular complications usually occur in patients with a history of pelvic radiation therapy or pelvic surgery [36]. Pelvic radiation appears to increase the risk of AUS-related complications, regardless of whether it is administered before or after AUS insertion [37]; nevertheless, some studies have not observed significant differences in the rate of intraoperative complications [38]. Radiation damages blood vessels, resulting in occlusion and thrombosis, and neovascularization; this leads to fibroblast proliferation and scar formation. Vascular injuries most frequently occur during the placement of the reservoir balloon, given the proximity of major vascular structures to the external inguinal ring. The reservoir balloon is placed after the different anatomical spaces have been dissected blindly. The external iliac vein distance from the inguinal ring is 2.5 to 4 cm at a 20- to 60-degree lateral measurement from the inguinal ring [39]. Careful dissection is essential and deep lateral dissection to the inguinal ring should be avoided [40]. In addition, to avoiding complications, a reservoir balloon can be placed in various locations, including the retroperitoneum, the prevesical space or the suprafascial abdominal level. Radiation-induced vascular changes and tissue fibrosis require an even more careful surgical technique to avoid intraoperative vascular complications.

Haematomas may appear in the scrotum, perineum or vulva 1–3 days after the placement of an AUS due to perioperative bleeding. This is especially likely if the patient is taking anticoagulants, has vascular disease or a coagulation disorder [41]. Poor microcirculation due to cardiovascular comorbidities requiring anticoagulation has been proposed as a risk factor [42]. These haematomas may be accompanied by inflammation, pain, altered sensitivity or contribute to pump migration. Mild cases resolve with rest, local cold application and medical follow-up. However, significant bleeding may require transfusion or additional surgical intervention to control the haemorrhage. Large hematomas can become infected, which can lead to infection of the UAS components. Surgical treatment is recommended in these cases to prevent complications, such as migration or device failure [36].

## 5. Conclusions

Although perioperative complications in minimally invasive pelvic floor surgery are uncommon, injury to adjacent organs such as the vagina, ureters, bladder, intestine, or particularly vascular injuries can pose a surgical challenge as they can endanger the patient’s life. Dissection of the sacral promontory during SC requires a precise and detailed understanding of pelvic anatomy and adequate preoperative planning.

The ability to anticipate anatomical variations, vascular proximity, and fixation-related challenges is critical for optimizing surgical outcomes. Minimally invasive procedures, when integrated with advanced imaging modalities and tailored surgical strategies, foster a safer operative environment and may contribute to enhanced postoperative recovery and patient satisfaction.

## Figures and Tables

**Table 2:** Management Strategies for Presacral Vascular Lesions Based on Clinical Presentation.

Clinical Scenario	Treatment Options	Considerations/Risks
Hemodynamically stable with visible bleeding vessel	Direct hemostasis using bipolar energy Surgical clip placement	Risk of exacerbated bleeding due to vessel retraction during coagulation
Hemodynamically stable with inaccessible vessel	Orthopedic tacks Bone wax Sutures	Mechanical alternatives to direct coagulation
Minor bleeding or controlled lesion	Conservative management with topical hemostatic agents (Surgicel^®^ (Ethicon, Inc., Somerville, NJ, USA), Fibrillar™ (Ethicon, Inc., Somerville, NJ, USA), FloSeal^®^ (Baxter Healthcare Corporation, Deerfield, IL, USA))	Effective in cases where invasive intervention is not required
Major vascular injury or hemodynamic instability	Hemostatic sutures Conversion to open surgery	Requires more aggressive surgical intervention

## Supplementary Materials

The following supporting information can be downloaded at https://www.mdpi.com/article/10.3390/jcm14196740/s1, Table S1: Characteristics of the studies included in the narrative review.

## Abbreviations

The following abbreviations are used in this manuscript:

SC	Sacrocolpopexy
RSC	Robotic sacrocolpopexy
LSC	Laparoscopic sacrocolpopexy
AUS	Artificial urinary sphincter
ICG	Indocyanine green
IVC	Inferior vena cava
3D	Three dimensions
CT	Computed tomography
MRI	Magnetic resonance imaging
NA	Not available
LLS	Laparoscopic lateral suspension
R	Restrospective study
CR	Case report
RCT	Randomized controlled trial
OP	Observational prospective
SR	Systematic review

## References

[1] Paraiso M.F.R., Jelovsek J.E., Frick A., Chen C.C.G., Barber M.D. (2011). Laparoscopic Compared with Robotic Sacrocolpopexy for Vaginal Prolapse. Obstet. Gynecol..

[2] Nosti P.A., Umoh-Andy U., Kane S., White D.E., Harvie H.S., Lowenstein L., Gutman R.E. (2014). Outcomes of Abdominal and Minimally Invasive Sacrocolpopexy. Female Pelvic Med. Reconstr. Surg..

[3] Ferrando C.A., Paraiso M.F.R. (2019). A Prospective Randomized Trial Comparing Restorelle Y Mesh and Flat Mesh for Laparoscopic and Robotic-Assisted Laparoscopic Sacrocolpopexy. Female Pelvic Med. Reconstr. Surg..

[4] Antosh D.D., Grotzke S.A., McDonald M.A., Shveiky D., Park A.J., Gutman R.E., Sokol A.I. (2012). Short term outcomes of robotic versus conventional laparoscopic sacral colpopexy. Female Pelvic Med. Reconstr. Surg..

[5] Yang J., He Y., Zhang X., Wang Z., Zuo X., Gao L., Hong L. (2021). Robotic and laparoscopic sacrocolpopexy for pelvic organ prolapse: A systematic review and meta-analysis. Ann. Transl. Med..

[6] Nygaard I.E., McCreery R., Brubaker L., Connolly A., Cundiff G., Weber A.M., Zyczynski H. (2004). Pelvic Floor Disorders Network. Abdominal sacrocolpopexy: A comprehensive review. Obstet. Gynecol..

[7] Siddiqui N.Y., Geller E.J., Visco A.G. (2012). Symptomatic and anatomic 1-year outcomes after robotic and abdominal sacrocolpopexy. Am. J. Obstet. Gynecol..

[8] Anger J.T., Mueller E.R., Tarnay C., Smith B., Stroupe K., Rosenman A., Brubaker L., Bresee C., Kenton K. (2014). Robotic compared with laparoscopic Sacrocolpopexy. Obstet. Gynecol..

[9] Unger C.A., Paraiso M.F.R., Jelovsek J.E., Barber M.D., Ridgeway B. (2014). Perioperative adverse events after minimally invasive abdominal sacrocolpopexy. Am. J. Obstet. Gynecol..

[10] Gutzeit O., Lauterbach R., Loberman Z., Sachner R., Karram T., Lowenstein L. (2020). Laparoscopic sacrocolpopexy complication: Ilio-femoral deep vein thrombosis. Eur. J. Obs. Gynecol. Reprod. Biol..

[11] Mahoney C., Scott G., Dwyer L., Reid F., Ward K., Smith A., Kearney R. (2019). Laparoscopic sacrocolpopexy posthysterectomy: Intraoperative feasibility and safety in obese women compared with women of normal weight. Int. Urogynecol. J..

[12] Turner L., Lavelle E., Lowder J.L., Shepherd J.P. (2016). The impact of obesity on intraoperative complications and prolapse recurrence after minimally invasive Sacrocolpopexy. Female Pelvic Med. Reconstr. Surg..

[13] Zhao Y., St-Martin B. (2020). Robotic-assisted laparoscopic sacrocolpopexy: Initial Canadian experience. Can. Urol. Assoc. J..

[14] Barakat B., Franke K., Hijazi S., Schakaki S., Gauger U., Hasselhof V., Vögeli T.A. (2020). A systematic review and meta-analysis of clinical and functional outcomes of artificial urinary sphincter implantation in women with stress urinary incontinence. Arab. J. Urol..

[15] Peyronnet B., Vincendeau S., Tondut L., Bensalah K., Damphousse M., Manunta A. (2016). Artificial urinary sphincter implantation in women with stress urinary incontinence: Preliminary comparison of robot-assisted and open approaches. Int. Urogynecol. J..

[16] Yip M.J., Jhamb A., Goad J.R. (2015). Case report of deep vein thrombosis caused by artificial urinary sphincter reservoir compressing right external iliac vein. Urol Ann..

[17] Lazarou G., Rahimi S., Cui N., Zormpa M. (2011). Variant iliocaval confluence discovered during sacrocolpopexy. Obstet. Gynecol..

[18] Giraudet G., Protat A., Cosson M. (2018). The anatomy of the sacral promontory: How to avoid complications of the sacrocolpopexy procedure. Am. J. Obstet. Gynecol..

[19] Wieslander C.K., Rahn D.D., McIntire D.D., Marinis S.I., Wai C.Y., Schaffer J.I., Corton M.M. (2006). Vascular anatomy of the presacral space in unembalmed female cadavers. Am. J. Obstet. Gynecol..

[20] Berger A.A., Abramowitch S., Moalli P.A. (2019). 3D vascular anatomy of the presacral space: Impact of age and adiposity. Int. Urogynecol. J..

[21] Carracedo-Calvo D., Pereira-Rodriguez N., Moscatiello P., Jerez-Izquierdo T., Meilán-Hernández E., Toledo-Jimenez M., Hernández- Bermejo I., Gimbernat-Diaz H., Sánchez- Encinas M. (2024). Robotic sacrocolpopexy for the treatment of pelvic organ prolapse in elderly women: Comparative analysis of safety and efficacy versus younger women. Actas Urol. Esp..

[22] Crisp C.C., Herfel C.V., Pauls R.N., Westermann L.B., Kleeman S.D. (2016). Critical Anatomy Relative to the Sacral Suture: A Postoperative Imaging Study After Robotic Sacrocolpopexy. Female Pelvic Med. Reconstr. Surg..

[23] Degirmenci Y., Schepers M., Steetskamp J., Hasenburg A., Skala C. (2023). Three-dimensional vs. two-dimensional endoscopic approach in urogynecology: A retrospective cohort study of laparoscopic sacrocolpopexy. J. Obs. Gynaecol. Res..

[24] Jun H.S., Lee N., Gil B., Jang Y., Yu N.K., Jung Y.W., Yun B.S., Kim M.K., Won S., Seong S.J. (2024). Intraoperative Fluorescent Navigation of the Ureters, Vessels, and Nerves during Robot-Assisted Sacrocolpopexy. J. Pers. Med..

[25] Popov A., Klyushnikov I., Fedorov A., Koval A., Tyurina S., Idashkin A. (2022). Sacrocolpopexy: Anatomical landmarks, clinical appliance and 3-year outcomes. Gynecol. Pelvic Med..

[26] Dubuisson J.B., Chapron C. (1998). Laparoscopic iliac colpo-uterine suspension for the treatment of genital prolapse using two meshes: A new operative laparoscopic approach. J. Gynecol. Surg..

[27] Kumbasar S., Salman S., Sogut O., Gencer F.K., Bacak H.B., Tezcan A.D., Timur G.Y. (2023). Uterine-sparing laparoscopic lateral suspension in the treatment of pelvic organ prolapse. J. Obs. Gynaecol. Res..

[28] Miele G.M., Marra P.M., Cefalì K., Venturella R., Di Carlo C., Zullo F. (2021). A New Combined Laparoscopic-Vaginal Lateral Suspension Procedure for the Treatment of Pelvic Organ Prolapse. Urology.

[29] Dällenbach P. (2022). Laparoscopic Lateral Suspension (LLS) for the Treatment of Apical Prolapse: A New Gold Standard?. Front. Surg..

[30] Boukerrou M., Orazi G., Nayama M., Boodhun R., Crépin G., Cosson M. (2003). Promontofixation procedure: Use of non-absorbable sutures or Tackers?. J. Gynecol. Obstet. Biol. La Reprod..

[31] Papp S.B., Gaitonde S., Baig S., Zimmern P.E. (2023). Hemorrhage Occluder™ Pin to control life-threatening bleeding during the removal of an infected sacrocolpopexy mesh: A case report. Case Rep. Womens Health..

[32] Hokenstad E.D., Occhino J.A. (2020). Management of presacral bleeding. Int. Urogynecol. J..

[33] Panico G., Mastrovito S., Arrigo D., Riccetti C., Campagna G., Scambia G., Ercoli A. (2025). Laparoscopic management of presacral retroperitoneal haematoma after sacrocolpopexy. Facts Views Vis Obgyn..

[34] Peyronnet B., O’Connor E., Khavari R., Capon G., Manunta A., Allue M., Hascoet J., Nitti V.W., Gamé X., Gilleran J. (2019). AMS-800 Artificial urinary sphincter in female patients with stress urinary incontinence: A systematic review. Neurourol Urodyn..

[35] Lin L., Sun W., Guo X., Zhou L. (2022). Artificial Urinary Sphincter Is Better Than Slings for Moderate Male Stress Urinary Incontinence with Acceptable Complication Rate: A Systematic Review and Meta-Analysis. Front. Surg..

[36] Frazier R.L., Jones M.E., Hofer M.D. (2024). Artificial Urinary Sphincter Complications: A Narrative Review. J. Clin. Med..

[37] DeLay K.J., Haney N.M., Chiang J., Stewart C., Yafi F.A., Angermeier K., Wood H., Boone T., Kavanagh A.G., Gretzer M. (2018). Comparison of adjuvant radiation therapy before or after artificial urinary sphincter placement: A multi-institutional, retrospective analysis. Urology.

[38] Mamane J., Sanchez S., Lellouch A.G., Gaillard V., Poussot B., Tricard T., Saussine C., Brierre T., Game X., Beraud F. (2022). Impact of radiation therapy on artificial urinary sphincter implantation in male patients: A multicenter study. Neurourol. Urodyn..

[39] Henry G., Hsiao W., Karpman E., Bella A.J., Carrion R., Jones L., Christine B., Eisenhart E., Cleves M.A., Kramer A. (2014). A guide for inflatable penile prosthesis reservoir placement: Pertinent anatomical measurements of the retropubic space. J. Sex. Med..

[40] Cayetano-Alcaraz A.A., Yassin M., Desai A., Tharakan T., Tsampoukas G., Zurli M., Minhas S. (2021). Penile implant surgery-managing complications. Fac. Rev..

[41] Khouri R.K., Ortiz N.M., Dropkin B.M., Joice G.A., Baumgarten A.S., Morey A.F., Hudak S.J. (2021). Artificial Urinary Sphincter Complications: Risk Factors, Workup, and Clinical Approach. Curr. Urol. Rep..

[42] Kretschmer A., Buchner A., Grabbert M., Stief C.G., Pavlicek M., Bauer R.M. (2016). Risk factors for artificial urinary sphincter failure. World J. Urol..

