# Supplementary material for: Critical Care and Surgical Management of Vascular Complications in Minimally Invasive Urological Reconstructive Surgery
Source: J Clin Med. 2025 Sep 24;14(19):6740. doi: 10.3390/jcm14196740 (PMC12524609; doi:10.3390/jcm14196740)
Supplement: Supplementary file 1 [file jcm-14-06740-s001.zip › jcm-3847208-supplementary.pdf]

Table S1-Supplementary material: - Characteristics of the studies included in the narrative review.

Study	Design	n	Surgical Approach	Vascular injury (n)	Transfusion rate (n)	Bowel injury (n)	Ureteral injury (n)	Bladder injury (n)
COMPLICATION INCIDENCE SC								
**Paraiso et al. [1]**	RCT	68	LSC and RSC	NA	NA	1	NA	4
**Nosti et al. [2]**	R	535	RSC and LSC	1	0	4	0	10
**Antosh et al. [4]**	R	88	RSC and LSC	NA	3	0	0	4
**Siddiqui et al. [7]**	R	125	RSC	1	1	0	NA	2
**Anger JT et al. [8]**	RCT	78	RSC and LSC	2	NA	2	NA	NA
**Unger et al. [9]**	R	370	RSC and LSC	3	2	6	0	4
**Gutzeit, O. et al. [10]**	CR	1	LSC	1	0	NA	NA	NA
**Mahoney, C et al. [11]**	R	119	LSC	0	0	1	1	4
**Turner L, et al. [12]**	R	556	LSC and RSC	NA	1	4	2	16
**Zhao and Martin. [13]**	R	47	RSC	0	1	0	1	2
TECHNIQUES FOR PREVENTING AND TREATING COMPLICATIONS IN SC								
**Berger AA et al. [20]**	R	107	SC	NA	NA	NA	NA	NA
**Degirmenci [23]**	R	71	LSC	0	0	2	0	1
**Jun HS et al. [24]**	R	15	RSC	NA	0	0	NA	0

**Kumbasar et al. [27]**	OP	62	LLS	0	0	0	NA	0
**Papp. [31]**	CR	1	SC	1	1	0	0	0
**Panico G. [33]**	CR	1	LSC	1	0	0	0	0
ARTIFICIAL URINARY SPHINCTER								
**Barakat et al. [14]**	SR	193	AUS	NA	NA	NA	NA	33
**Peyronnet et al. [15]**	R	8	AUS	0	0	NA	NA	1
**Yip MJ, et al. [16]**	CR	1	AUS	1	0	0	0	0

* SC: Sacrocolpopexy; LSC: laparoscopic sacrocolpopexy; RSC: robotic sacrocolpopexy; NA: not available; R: retrospective study; CR: case report; RCT: randomized controlled trial; OP: Observational prospective; LLS; Laparoscopic lateral suspension; SR: systematic review; AUS: artificial urinary sphincter
